# Purine metabolism disruption links pathology and cognitive decline in Alzheimer’s disease

**DOI:** 10.1172/jci.insight.205511

**Published:** 2026-10-22

**Authors:** Xiaohang Qian, Zhongxuan Wang, Siyue Chen, Yang Xue, Yawen Yang, Peijing Cui, Miao Zhang, Huidong Tang

**Affiliations:** 1 Department of Geriatrics, Medical Center on Aging, Ruijin Hospital,; 2 Department of Neurology and Institute of Neurology, Ruijin Hospital, and; 3 Department of Rehabilitation Medicine, Ruijin Hospital, Shanghai Jiao Tong University School of Medicine, Shanghai, China.; 4 Department of Nuclear Medicine, Ruijin Hospital, Shanghai Jiao Tong University, Shanghai, China.

**Keywords:** Aging, Metabolism, Neuroscience, Alzheimer disease

## Abstract

Purine metabolism dysregulation has been implicated in Alzheimer’s disease (AD), but its temporal dynamics and mechanistic links to core pathologies remain unclear. This translational study integrated a longitudinal preclinical AD mouse model and clinical cohort investigations to define the role of purine metabolism in AD progression. In APPswe/PSEN1dE9 (APP/PS1) mice, serum-untargeted metabolomics identified purine metabolism as the most notably altered pathway, with metabolite levels (e.g., adenine, adenosine) shifting dynamically across disease stages alongside increasing amyloid-β (Aβ) burden. In the pathology-defined clinical cohort, targeted analysis revealed that serum uric acid and allantoin were specifically depleted in the Aβ^+^ cognitively impaired (CI) group and correlated strongly with higher brain Aβ load, increased neuroinflammation, and poorer cognitive scores. Formal mediation analysis elucidated a plausible mechanistic cascade: the association of uric acid/allantoin with cognitive impairment was primarily mediated by Aβ pathology. Two-year longitudinal data further suggest that changes in specific purine metabolites might predict the rate of cognitive decline. Allantoin supplementation reduced Aβ pathology through promoting microglial phagocytosis of Aβ in APP/PS1 mice. Our findings identified purine metabolism as a key hub connecting Aβ pathology, neuroinflammation, and clinical progression in AD.

## Abstract 2

<strong>Purine Metabolism Disruption</strong> <strong>as Candidate Biomarkers Linking Aβ Pathology, Neuroinflammation, and Cognitive Decline in Alzheimer’s Disease</strong>

## Introduction

Alzheimer’s disease (AD), the most prevalent cause of dementia, represents a monumental and growing public health challenge worldwide (1). The core neuropathological hallmarks of AD — extracellular amyloid-β (Aβ) plaques and intracellular neurofibrillary tangles composed of hyperphosphorylated Tau (2, 3). They have been the focus of intense research for decades, leading to the recent advent of disease-modifying therapies targeting Aβ (4). However, the clinical outcomes of these therapies remain modest and variable, underscoring the complex, multifactorial nature of AD pathogenesis beyond the simple accumulation of misfolded proteins (5, 6). This complexity is reflected in the persistent failures of numerous late-stage clinical trials (6). These highlight a critical need to identify earlier, more sensitive biomarkers and to elucidate the downstream and parallel pathological cascades.

In recent years, the conceptualization of AD has evolved beyond a purely “brain-limited” proteinopathy to acknowledge considerable contributions from systemic metabolic dysregulation and neuroimmune dysfunction (7–9). Metabolic alterations are increasingly recognized not merely as secondary epiphenomena but as active drivers of disease progression. For instance, brain insulin resistance, mitochondrial dysfunction, and lipid metabolism disturbances are well documented in AD (9–11). They are thought to contribute to bioenergetic failure and oxidative stress, creating a vulnerable neuronal environment. Large-scale metabolomic profiling studies in cerebrospinal fluid (CSF), plasma, and brain tissue from patients with AD and animal models have consistently revealed widespread metabolic disturbances, implicating pathways involved in energy metabolism, amino acid metabolism, and lipid metabolism (8, 12, 13). Among these, perturbations in purine metabolism have emerged as a recurring, yet mechanistically underexplored, signature.

Purine nucleotides (e.g., ATP, ADP) and their catabolites (e.g., adenosine, inosine, hypoxanthine, uric acid) are fundamental to cellular bioenergetics, nucleic acid synthesis, and signal transduction (14). In the brain, ATP is the primary energy currency, and it is controlled degradation through the purine catabolic pathway (14). In addition, it is crucial for maintaining energy homeostasis and generating important signaling molecules like adenosine (15). They modulate neurotransmission and synaptic plasticity as well as neuroinflammation through purinergic receptors (15). Notably, several lines of evidence point to a dysregulation of this pathway in AD (16–18). Postmortem AD brain studies have shown altered levels of purine metabolites (16, 17). Plasma metabolomic analyses have reported changes in levels of metabolites such as uric acid in individuals with mild cognitive impairment (MCI) and AD (19, 20). Uric acid, the terminal oxidation product of purine metabolism, possesses potent antioxidant properties (21). A series of studies have reported an association between higher levels of uric acid and the delay of cognitive decline in AD (22). However, previous studies lacked comprehensive analysis of the overall characteristics of purine metabolism as well as integrated analysis with the overall pathological markers of AD. This may obscure the true biological relationship between systemic purine metabolism and core AD pathology. Crucially, the temporal dynamics of purine metabolic changes relative to Aβ and Tau accumulation, their specific associations with in vivo measures of pathology and neuroinflammation, and their mechanistic link to cognitive impairment remain poorly defined.

To address these critical gaps, this study integrated preclinical and clinical study phases. In the preclinical phase, we conducted cross‑sectional and longitudinal serum metabolomics alongside quantitative analysis of cerebral Aβ pathology in APPswe/PSEN1dE9 (APP/PS1) mice, aiming to identify purine metabolites associated with disease features and progression. In the clinical phase, we first performed a cross‑sectional comparison of serum purine metabolic profiles between Aβ^+^ cognitively impaired (CI) and Aβ^–^ cognitively unimpaired (CU) individuals, and we examined their correlations with pathological burden and cognitive function. Furthermore, using 2‑year longitudinal data, we analyzed dynamic changes in serum purine metabolism in relation to plasma pathological biomarkers and cognitive decline to assess their prognostic potential. Finally, a reverse validation was conducted on APP/PS1 mice by using specific purine metabolites. This work aims to position purine metabolism not just as a biomarker source but as a key integrative node in the AD pathological network, connecting metabolic stress, proteinopathy, and neuroinflammation.

## Results

### Purine metabolism represented the most marked metabolic alteration distinguishing APP/PS1 from WT mice.

Consistent with the established phenotype, 8-month-old APP/PS1 mice exhibited evident Aβ deposition in the cortex and hippocampus, which was absent in WT littermates (Figure 1A). Untargeted serum metabolomics revealed obvious dysregulation in APP/PS1 mice, with 14 metabolites downregulated and 26 metabolites upregulated compared with WT controls (Figure 1B). Pathway enrichment analysis indicated that these differential metabolites were primarily associated with purine metabolism, lysine degradation, glycerophospholipid metabolism, linoleic acid metabolism, and taurine and hypotaurine metabolism (Figure 1C). Among these, the purine metabolism pathway was the most significantly altered. Targeted analysis of purine-related metabolites demonstrated significant upregulation of adenine, adenosine, guanosine, hypoxanthine, and inosine in APP/PS1 mouse serum (Figure 1, D–M). These findings identify purine metabolism disruption as a characteristic metabolic alteration in this AD mouse model.

### Purine metabolism was a hallmark metabolic alteration that persists across disease stages in APP/PS1 mice.

As expected, APP/PS1 mice displayed a progressive increase in cerebral Aβ plaque burden and brain atrophy from 5 to 15 months of age (Figure 2, A–C). Serum metabolomic profiling across these 3 time points (5, 10, and 15 months) revealed distinct metabolic trajectories. Principal component analysis (PCA) showed clear separation of metabolic profiles among the different age groups, indicating obvious shifts in the metabolome with disease progression (Figure 2D). Pairwise comparisons identified 112, 39, and 104 differential metabolites between the 10- versus 5-month, 15- versus 10-month, and 15- versus 5-month groups, respectively (Figure 2E). Purine metabolism emerged as the most significantly enriched pathway across all longitudinal comparisons (Figure 2, F–H). Detailed analysis showed that the abundance of adenine, adenosine 5’-phosphosulfate, adenosine monophosphate (AMP), adenosine, and guanosine increased progressively with age in APP/PS1 mice (Figure 2, I–M). In contrast, the levels of deoxyinosine, xanthine, hypoxanthine, deoxyguanosine, and inosine peaked at 10 months of age (Figure 2, N–R). While uric acid levels showed no significant age-related changes, and allantoin abundance decreased progressively with age in APP/PS1 mice (Figure 2, S and T). These data suggest that purine metabolism was not only disrupted in AD onset but was also dynamically coupled to disease progression.

### Altered purine metabolism profile and correlations with pathological markers in the Alzheimer’s Disease Immunization and Microbiota Initiative study Cohort (ADIMIC).

We quantitatively profiled serum purine metabolites in 41 Aβ^–^ CU and 62 Aβ^+^ CI participants from the ADIMIC. The CI group exhibited the canonical AD signature: obviously decreased Mini-Mental State Examination (MMSE) and Montreal Cognitive Assessment (MoCA) scores, elevated brain amyloid deposition and microglial activation, alongside peripheral biomarkers indicative of AD pathology (reduced plasma Aβ42/40 ratio and elevated glial fibrillary acidic protein [GFAP], neurofilament light chain [NFL], and phosphorylated Tau-181 [p-Tau181] levels) (Table 1). Of the 8 purine metabolites detected, adenine, AMP, guanosine, inosine, xanthine, and hypoxanthine showed no significant difference between CU and CI groups (Figure 3, A–F). Strikingly, uric acid and allantoin levels were significantly lower in the CI group, compared with CU group (Figure 3, G and H). Correlation analysis revealed that serum allantoin level was positively correlated with plasma Aβ42, Aβ40, Aβ42/40 ratio, MMSE, and MoCA scores and negatively with global AV45 SUVR (Figure 3I). Similarly, uric acid level was positively correlated with plasma Aβ42, Aβ40, and MMSE scores and negatively with global AV45 SUVR (Figure 3I). Thus, uric acid and alantoin were key differential purine metabolites in the human AD cohort, showing obvious associations with both Aβ pathology and cognitive status.

To elucidate the mechanistic link between these metabolites and cognition, we performed mediation analysis. The effect of uric acid on MMSE scores was fully mediated by AV45 SUVR and plasma Aβ42 (Figure 3, J–L). Similarly, the effect of allantoin on MMSE scores was fully mediated by AV45 SUVR and plasma Aβ42 levels and was partially mediated by the Aβ42/40 ratio (Figure 3, M–O). Its effect on MoCA scores was fully mediated by AV45 SUVR and partially mediated by Aβ42 and Aβ42/40 (Figure 3, P–R). In summary, both uric acid and allantoin appeared to influence cognitive impairment primarily through their association with Aβ pathology.

### Uric acid and allantoin were linked to Aβ pathology via distinct neuroinflammatory pathways.

Given prior evidence linking purine metabolism to neuroinflammation in AD, we investigated correlations with in vivo (DPA714 PET) and peripheral (GFAP) inflammatory markers. Serum uric acid correlated negatively with plasma GFAP but not with global DPA714 SUVR (Figure 4, A and B). Conversely, serum allantoin levels showed a negative correlation with global DPA714 SUVR but not with plasma GFAP (Figure 4, C and D). Brain region correlations were detailed in Figure 4, E and F. Since glial activation was closely tied to Aβ pathology, we next tested if their association with Aβ was mediated by neuroinflammation. Mediation analysis indicated that the association between uric acid and global AV45 SUVR was fully mediated by plasma GFAP, while its associations with plasma Aβ42 were partially mediated by GFAP (Figure 4, G–I). In addition, the association between allantoin and global AV45 SUVR was fully mediated by DPA714 SUVR, but not by plasma Aβ42 or Aβ42/40 (Figure 4, J–L). These results suggested that uric acid and allantoin were associated with Aβ pathology through distinct neuroinflammatory pathways, implicating different glial cell types.

### Longitudinal changes of purine metabolism and correlation with pathological progression.

We analyzed 2-year longitudinal data from a subset of the ADIMIC (7 CU and 19 CI). The CU group remained cognitively stable with no significant change in AD biomarkers, whereas the CI group showed obvious cognitive decline and biomarker progression (Table 2). In the CI group, serum AMP and uric acid levels significantly decreased, while guanosine levels increased over the 2-year period (Figure 5, A, D, and G). In the CU group, only adenine levels showed a significant decrease (Figure 5B). Inosine, xanthine, hypoxanthine, and allantoin levels remained stable over time in both groups (Figure 5, C, E, F, and H). Importantly, the longitudinal change of ΔAMP was negatively correlated with ΔGFAP and was positively correlated with ΔAβ42/40. In addition, Δguanosine was negatively correlated with ΔMMSE and ΔMOCA, and was positively correlated with ΔAβ42/40 (Figure 5I). These findings suggested that changes in AMP and guanosine may predict the progression of specific pathological features (Aβ42/40, GFAP), while changes in guanosine may predict the rate of cognitive decline.

### Allantoin supplementation reduced Aβ pathology through promoting microglial phagocytosis of Aβ in APP/PS1 mice.

To investigate the causal role of allantoin in AD pathogenesis, we performed an intervention study in APP/PS1 mice. Mice at 8 months of age were orally administered allantoin or vehicle control for 2 months. Immunofluorescence staining for Aβ revealed that chronic allantoin supplementation obviously decreased the Aβ plaque load compared with vehicle-treated APP/PS1 controls (Figure 6, A and B). To elucidate the underlying mechanism, we costained brain sections with Iba-1 and Cd68. Notably, quantitative analysis showed a higher proportion of Cd68^+^/Iba-1 cells around the plaque in the allantoin-treated group (Figure 6, C and D), suggesting an enhanced microglial phagocytic phenotype. Collectively, these intervention data indicated that allantoin supplementation reduced Aβ burden by augmenting the phagocytic capacity of plaque-associated microglia.

## Discussion

This study employed an integrated translational approach to systematically elucidate the role of purine metabolism in AD pathophysiology. Our principal findings were 3-fold. First, we identified purine metabolism as the most significantly perturbed metabolic pathway in APP/PS1 mice, a disruption that exhibits dynamic, stage-specific alterations coupled with Aβ deposition progression. Second, we translated these findings to a clinical cohort, pinpointing 2 specific purine catabolites — uric acid and allantoin — as obviously depleted in Aβ^+^ CI individuals. These metabolites showed robust correlations with in vivo Aβ burden, neuroinflammation, and cognitive function. Third, through formal mediation analyses, we constructed a plausible mechanistic model wherein uric acid and allantoin were linked to cognitive impairment primarily through Aβ pathology. More importantly, regulation of Aβ pathology appeared to be modulated by distinct neuroinflammatory pathways associated with each metabolite. These convergent lines of evidence position purine metabolism, particularly its terminal oxidation products, as a critical nexus linking Aβ pathology, neuroinflammation, and cognitive decline in AD.

Our finding of widespread purine metabolite dysregulation in the APP/PS1 mice serum aligns with and significantly extends prior metabolomic studies in AD models and patients. Earlier reports have documented alterations in purine nucleosides and bases in the brain, CSF, or plasma of individuals with AD and of transgenic mice (23–25). For instance, increased levels of xanthine and inosine have been observed in AD brains, potentially reflecting increased ATP turnover and energetic stress (23). Our study confirmed these alterations in serum and, more importantly, provided a longitudinal map of their evolution. The progressive increase in precursors like adenine and AMP alongside the decline in the antioxidant metabolite allantoin suggests a shift in purine metabolism that may favor biosynthesis and oxidative stress over catabolism and clearance. This pattern provides a metabolic context for the well-documented energetic deficit and oxidative damage in AD (26). The decline in deoxyinosine, xanthine, and hypoxanthine between 10 and 15 months was particularly noteworthy. We speculated that this represents a metabolic exhaustion, where the initial compensatory increase in nucleotide turnover seen at 10 months is no longer sustainable during the end-stage of severe amyloid deposition and neuronal loss. This bimodal trajectory is characteristic of failing compensatory mechanisms in chronic diseases.

In ADIMIC, our data showed the specific and consistent reduction of serum uric acid of Aβ^+^ CI group. This was consistent with series previous epidemiological studies that uric acid is decreased in patients with AD and is a protective factor of cognition, may attribute to its antioxidant properties (27). In addition, serum uric acid level was closely related to the MMSE scores and the Aβ pathological marker. A recent study has found that the serum uric acid level was positively correlated with the levels of plasma Aβ42 and Aβ40, as well as the MMSE score, in patients with AD (28). This was consistent with the results of this study. However, in another study involving patients without dementia, it was found that serum uric acid level was not correlated with the level of Aβ load in the brain (29). This might be related to different stages of the disease. The most striking and novel translational finding was that allantoin was also decreased in Aβ^+^ CI group. The strong negative correlation between allantoin and global Aβ load was particularly intriguing, as allantoin is a nonenzymatic oxidation product of uric acid, often considered a marker of oxidative stress (30). Furthermore, preclinical study has also shown that allantoin has the ability to improve cognitive impairment in an Aβ1-42–induced AD model in rats (31). Its decrease, alongside uric acid, could paradoxically indicate an exhaustion of this oxidative pathway or a fundamental shift in purine catabolism preceding clinical onset.

The mediation analyses offered a substantial step beyond correlation, proposing a testable mechanistic hierarchy. The finding that the effects of uric acid and allantoin on cognition were largely mediated by Aβ pathology positions. This indicated that these metabolites were upstream to Aβ accumulation in the pathogenic cascade, rather than merely being secondary consequences. This was reinforced by the subsequent discovery that their links to Aβ pathology were themselves mediated by neuroinflammation — but in distinctly different ways. The pathway linking allantoin to Aβ via TSPO-PET signal (primarily reflecting microglial activation) suggests an involvement with innate immune responses in the brain (32). In contrast, the pathway linking uric acid to Aβ via plasma GFAP (a marker of astrocytic activation) implicates a different glial cell type (33). This dichotomy aligns with emerging knowledge about the specialized roles of microglia and astrocytes in Aβ clearance and neuroinflammation (33). However, the latest preclinical studies have shown that uric acid treatment can promote the phagocytosis of Aβ by microglia in 5×FAD mice (34). This might be related to the uric acid treatment leading to an increase in downstream allantoin. In this study, these intervention data indicated that allantoin supplementation can reduce Aβ burden by augmenting the phagocytic capacity of plaque-associated microglia. Overall, integrating our current research and previous studies suggests that purine metabolism might differentially regulate these cell types, offering avenues for targeted immunomodulation.

The longitudinal data, though from a modest sample, provide preliminary but crucial evidence for the prognostic utility of purine metabolites. The observed dynamic changes — decreasing AMP and uric acid, and increasing guanosine in progressive CI individuals — mirror and extend the cross-sectional findings. The predictive correlations between ΔGuanosine/ΔAβ42/40, ΔAMP/ΔGFAP, and ΔAMP/ΔAβ42/40 suggest that specific purine metabolites could serve as sensitive, dynamic blood-based indicators of ongoing Aβ pathology and astrocytosis, respectively. The strong negative correlation between ΔGuanosine and ΔMMSE and ΔMOCA positions this nucleoside as a potential predictor of near-term cognitive decline. These findings warrant validation in larger, independent longitudinal cohorts but highlight the promise of purine metabolites as progression biomarkers, complementing existing canonical markers.

Furthermore, we also discovered the differences in purine metabolism patterns between mice and humans. This might be related to the following factors. Unlike humans, mice possess the enzyme uricase, which further oxidizes uric acid to allantoin (35). In mice, uric acid is not the terminal metabolite. Therefore, the increase in early purine precursors (adenine, AMP) in mice likely reflects a massive metabolic “burst” of ATP catabolism. The inability to see a rise in allantoin in aging mice suggests that the antioxidant pathway is overwhelmed or declining. In humans (who lack uricase), uric acid and allantoin represent the “exhaustion” of the purine catabolic antioxidant system (35). The decrease in uric acid and allantoin we observed in Aβ^+^ patients may indicate a consumption of these antioxidants to combat chronic oxidative stress and neuroinflammation, rather than a simple accumulation of upstream metabolites.

This study has several limitations. First, the observational nature of the clinical cohort precludes definitive causal inference. While mediation analysis suggests pathways, reverse causality or confounding by unmeasured variables (e.g., gut microbiota alterations affecting purine metabolism) cannot be ruled out (36). Second, the sample size for the longitudinal human analysis was small, limiting the statistical power and generalizability of those particular results. Third, we measured metabolites in serum. While this enhanced translational potential for a blood-based biomarker, it does not directly reflect CNS concentrations. The relationship between central and peripheral purine metabolism in AD is complex and requires further study using paired CSF-serum samples. Fourth, the APP/PS1 mice model primarily recapitulates Aβ pathology and cannot accurately reflect the Tau pathology. The role of purine metabolism in primary tauopathies remains to be explored. Finally, the specific enzymatic drivers of the observed purine metabolic shift — such as the potential downregulation of xanthine oxidase or uricase — were not investigated and represent an important target for future mechanistic studies.

In conclusion, this integrative multimodality study established purine metabolism as a key pathway dynamically engaged in the AD pathological continuum. Specific purine catabolites (particularly uric acid and allantoin) were robustly associated with in vivo Aβ pathology and neuroinflammation in patients with AD. And these metabolites influenced cognitive function through pathways mediated by core AD pathology.

## Methods

### Sex as biological variable

In preclinical animal study, both male and female mice were included with an equal sex ratio (1:1) in each experimental group. In a clinical cohort study, both male and female participants were also incorporated into study. Both sexes yielded similar outcomes.

### Study design

First, in a preclinical arm, we investigated purine metabolism disruptions in the APP/PS1 mouse model. We performed serum untargeted metabolomics on 8-month-old APP/PS1 mice alongside WT controls (*n* = 8 per group). This cross-sectional comparison was used to identify and distinguish the differences in purine metabolism between the disease group and the control group. Furthermore, to capture dynamic changes across disease progression, we analyzed serum metabolomics and brain Aβ load at 3 critical time points (5, 10, and 15 months) of APP/PS1 mice (*n* = 6 per group). These 3 time points represent presymptomatic, pathology-accumulating, and advanced pathological stages (37). This longitudinal approach in APP/PS1 mice aimed to identify progression-associated purine metabolites and establish their temporal coupling with Aβ deposition.

Second, to translate these findings to humans, we leveraged the ADIMIC. In ADIMIC, all participants have undergone neuropsychological assessment, plasma AD biomarkers detection (e.g., Aβ42/40, p-tau181, GFAP), and multimodal positron emission tomography (PET) imaging (amyloid-PET and TSPO-PET). In a cross-sectional analysis, we will compare serum purine metabolomic profiles between Aβ^+^ CI and Aβ^–^ CU participants, examining their correlations with in vivo pathology burden from plasma AD biomarkers and PET and from cognitive performance. More importantly, in a longitudinal subcohort followed for 2 years, we analyzed the dynamic changes in serum purine metabolism. We explored how these longitudinal metabolic shifts correlate with changes in plasma pathology biomarkers and the rate of cognitive decline, thereby assessing their prognostic potential.

Finally, a reverse validation was conducted on APP/PS1 mice by allantoin supplementation. Eight-month-old APP/PS1 mice (*n* = 4 per group) were administered allantoin (3 mg/kg) by oral administration via oral gavage for 8 weeks. Then, the pathology of Aβ plaque and microglia activation in the brain of APP/PS1 mice were detected.

### Preclinical animal study

#### Experimental animal.

In this study, APP/PS1 mice and WT C57BL/6J mice were generated by the Shanghai Model Organisms Center. Mice were housed under standard laboratory conditions in individually ventilated cages within a specific pathogen–free (SPF) facility. The housing environment was maintained at 22°C ± 2°C with 55% ± 5% relative humidity and a 12-hour light/dark cycle.

#### Drug administration.

Allantoin (MedChemExpress, HY-N0543) was dissolved in 10% Tween 80 solution. Based on the results of the previous studies (38), a medium dose (3 mg/kg) was administered by oral administration via oral gavage for 8 weeks, and samples were collected for subsequent experiments.

#### Untargeted metabolomic analysis of serum.

Serum metabolomic profiling was conducted at Shanghai Applied Protein Technology Co. Ltd (APTBIO) as previously described (39). In brief, metabolites were extracted by mixing 100 μL serum with 400 μL of chilled methanol/acetonitrile/water (2:2:1, v/v/v). Following vortexing and incubation on ice for 20 minutes, the mixture was centrifuged (14,000*g*, 20 minutes, 4°C). The supernatant was collected, vacuum-dried at 4°C, and reconstituted in 100 μL of 50% acetonitrile for analysis. Chromatographic separation was performed on an Agilent 1290 Infinity LC UHPLC system coupled to a Sciex TripleTOF 6600 mass spectrometer operating in both positive and negative ion modes. Raw MS data were processed using XCMS (Scripps Research Institute) for feature detection, alignment, and extraction. Metabolite identification was achieved by matching MS/MS spectra against an in-house library of authentic standards. Pooled quality control (QC) samples were prepared by mixing equal volumes of all serum samples and injected periodically throughout the LC-MS/MS run. System stability was rigorously evaluated by overlaying total ion current (TIC) chromatograms, performing PCA to confirm tight QC clustering, calculating pairwise Pearson correlations (R > 0.9), and assessing relative SDs. Multivariate control charts (MCC) and Hotelling’s T^2^ tests further verified that all samples remained within the 99% CI and the analytical process was free of marked drift. Data were normalized by total peak area and Pareto-scaled prior to statistical analysis. Collectively, these QC metrics confirmed excellent analytical reproducibility, ensuring that the observed metabolic differences reflected genuine biological variations rather than technical artifacts. Statistical analysis included PCA for pattern recognition and unpaired 2-tailed Student’s *t* test/1-way ANOVA for group comparisons. Metabolites with a variable importance in projection (VIP) score > 1 and *P* < 0.05 were considered significantly altered. Pathway enrichment analysis was performed using MetaboAnalyst based on the KEGG database (https://www.metaboanalyst.ca/) (40).

#### Immunofluorescence assay.

Immunofluorescence was conducted following established protocols (41, 42). Briefly, 30 μm–thick mouse brain sections were permeabilized in 0.5% Triton X-100 in PBS (PBS-T) for 30 minutes. Nonspecific binding was blocked with 10% goat serum in PBS-T for 1 hour at room temperature. Sections were then incubated overnight at 4°C with primary antibody targeting Aβ (4G8, 1:1,000, BioLegend, 800709), Iba1 (1:500, Abcam, ab5076), and Cd68 (1:500, Proteintech, 28058-1-AP). After washing, sections were incubated for 2 hours at room temperature with appropriate fluorescent secondary antibodies and DAPI. Following extensive washes in PBS-T, sections were mounted with anti-quench medium. Imaging was performed using an 3DHISTECH scanner (‌Pannoramic MIDI‌ II), and fluorescence intensity was quantified using ImageJ software (NIH).

### Clinical cohort study

#### Participant recruitment.

Participants were enrolled from the ADIMIC cohort, following previously described inclusion/exclusion criteria (43). Participants underwent comprehensive evaluation, including neuropsychological assessment, 18F-AV45 and 18F-DPA714 PET/MR imaging, serum targeted metabolomics, and plasma AD biomarker profiling. Based on clinical evaluation (Petersen criteria for MCI and 2011 NIA-AA criteria for AD dementia) and amyloid PET status, participants were classified into 2 groups: (a) Aβ^+^ CI group (clinical MCI/AD dementia with Aβ^+^ PET), and (b) Aβ^–^ CU group (normal cognition with Aβ^−^ PET) (44–46).

#### PET/MRI acquisition and processing.

All participants underwent 18F-AV45 and 18F-DPA714 PET/MR imaging. Procedures for image acquisition, visual assessment, and processing followed previously established protocols (43). Briefly, PET data were acquired 40–60 minutes after injection of 18F-AV45/18F-DPA714 simultaneously with structural MRI, which included 3D T1-MPRAGE, FLAIR, and T2-weighted sequences. Visual assessment of Aβ based on 18F-AV45 PET was performed independently by 2 certified nuclear medicine radiologists in accordance with international consensus guidelines for amyloid PET interpretation (47). For quantitative analysis, T1-weighted MRI scans were processed using FreeSurfer 6.0.0 for tissue segmentation and regional parcellation. 18F-DPA714 PET images were coregistered to the corresponding MRI. Standard uptake value ratios (SUVRs) were generated using cerebellar gray matter as the reference region. Regional 18F-DPA714 SUVR values were extracted from cortical regions of interest defined by the Desikan-Killiany atlas.

#### Measurements of plasma AD biomarkers.

Blood samples were collected from all participants and processed to obtain plasma. Plasma concentrations of Aβ40, Aβ42, GFAP, NFL, and p-Tau181 were quantified using the single-molecule array (Simoa) technology. The assays were performed with commercial kits in accordance with the manufacturer’s protocols. The Simoa p-Tau181 kit for p-Tau181 and the Simoa N4PE Advantage kit for Aβ40, Aβ42, GFAP, and NFL were specifically employed.

#### Serum purine metabolism detection.

To extract metabolites from the serum, 400 μL of cold methanol/acetonitrile (1:1, v/v) was added to simultaneously remove proteins and extract metabolites, followed by thorough vortexing. For absolute quantification, stable-isotope–labeled internal standard stock solutions were added to the extraction solvent. The mixture was transferred to a new centrifuge tube and centrifuged at 14,000*g* for 20 minutes at 4°C. The supernatant was collected and dried in a vacuum centrifuge. Prior to LC-MS analysis, the dried samples were reconstituted in 100 μL of acetonitrile/water (1:1, v/v) and centrifuged again under the same conditions for 15 minutes, and the final supernatant was injected. Analyses were conducted at APTBIO using a UHPLC system (1290 Infinity LC, Agilent Technologies) coupled to a QTRAP mass spectrometer (6500+, AB Sciex). The mass spectrometer was operated in positive/negative switching mode. Quantitative data were acquired using multiple reaction monitoring, with ion pairs provided in the attached file. Pooled QC samples were included to monitor system stability and reproducibility. Metabolites with a coefficient of variation (CV) < 30% in QCs were considered reproducibly measured. Data processing was performed using MultiQuant or Analyst software.

### Statistics

In preclinical animal study, the difference of continuous variables between the 2 groups were subjected to 2-tailed Student’s *t* test or rank-sum tests based on the data’s normal distribution. The difference of categorical variables between the 2 groups was analyzed by χ^2^ test. The difference of continuous variables between the 3 groups were subjected to Kruskal-Wallis test followed by Dunn’s multiple-comparison test or 1-way ANOVA followed by Tukey’s multiple-comparison test based on the normality of the data distribution. In a clinical cohort study, the difference of continuous variables between the 2 groups was calculated through analysis of covariance adjusting for age, sex, education, and APOE4 carrier status. The correlation analysis was calculated through partial correlation analysis adjusting for age, sex, education, and APOE4 carrier status. The mediation analysis was performed by PROCESS v3.2 for SPSS (version 25.0) adjusted for age, sex, education, and APOE4 carrier status. In the longitudinal cohort analysis, the paired *t* test was employed. The *P* < 0.05 was regarded as significantly altered.

### Study approval

All animal experiments received ethical approval from the Animal Ethics Committee of the Shanghai Model Organisms Center (permit no. 2022-0008) and were carried out in compliance with the institution’s ethical regulations as well as internationally accepted animal welfare standards. The human clinical cohort study was approved by the IRB of Ruijin Hospital, affiliated with Shanghai Jiao Tong University School of Medicine (permit no. 2021-46). Written informed consent was obtained from each participant prior to enrollment.

### Data availability

All numerical data plotted in the figures are accessible in the Supporting Data Values file; supplemental material available online with this article; https://doi.org/10.1172/jci.insight.205511DS1 The datasets generated and analyzed during the current study are available from the corresponding author on reasonable request, provided that the applicant includes a concise statement outlining the planned use of the requested data.

## Author contributions

Conceptualization, designing study and revising the manuscript were contributed by HT, MZ, and PC. Experimentation and acquisition of data were contributed by XQ and ZW. Analysis and interpretation of data were contributed by XQ and ZW. Technical and material support were contributed by SC, YX, and YY. Clinically relevant guidance was contributed by HT and XQ. Writing of the original paper was contributed by XQ and ZW. Supervision was contributed by HT, MZ, and PC. XQ and ZW are co-first authors of this work. XQ is listed as the first co-first author because of longer involvement in the project.

## Funding support

- Shanghai Sailing Program, grant 24YF2725800 to XQ.
- China Postdoctoral Science Foundation, grant 2025M772189 to XQ.
- National Natural Science Foundation of China, grant 82372073 to MZ.

## Conflict of interest

The authors have declared that no conflict of interest exists.

## Supplementary Material

Supporting data values

## Figures and Tables

**Figure 1:**
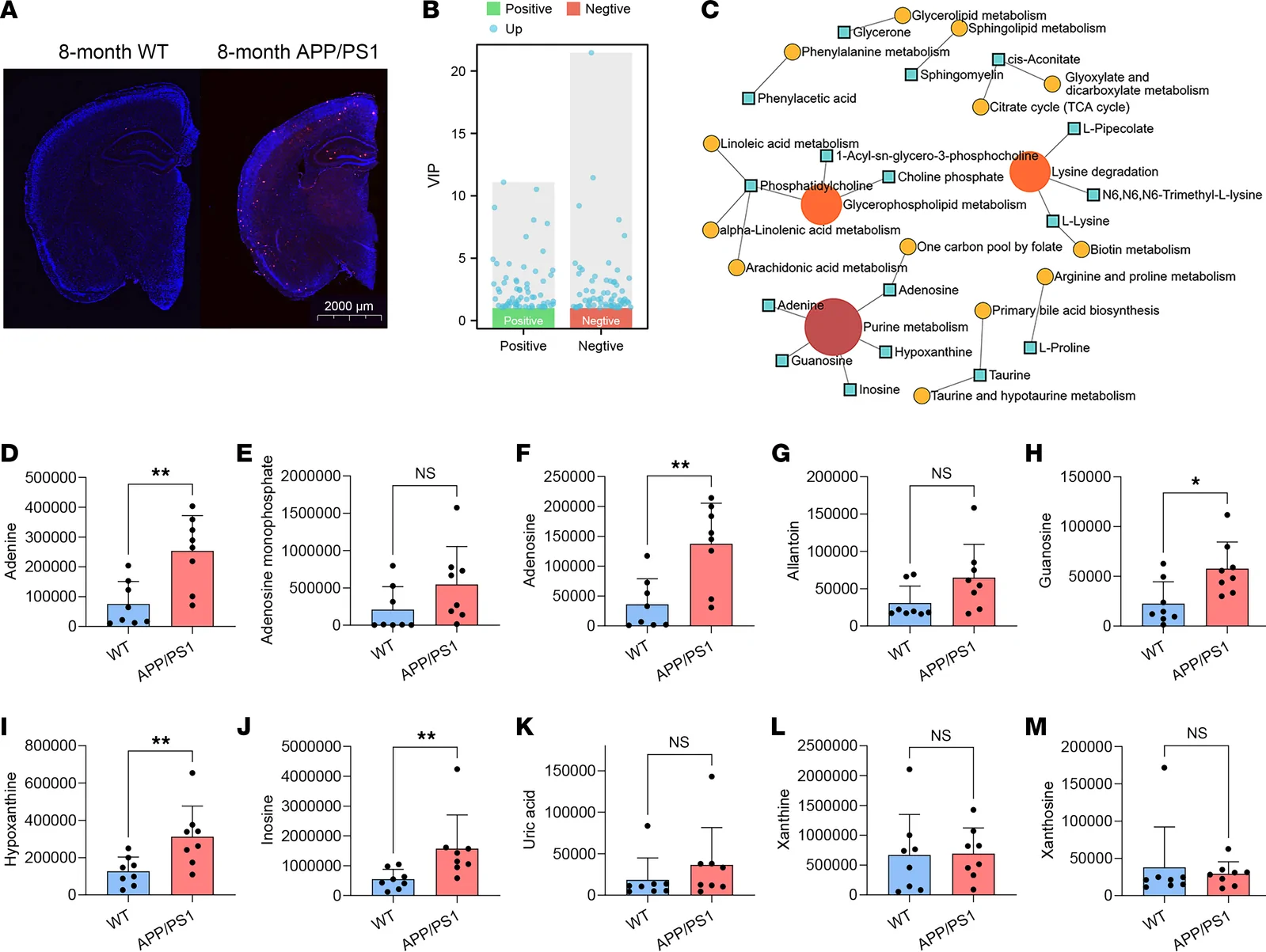
Metabolic profile differences in serum between WT and APP/PS1 mice. (**A**) Immunofluorescence of 4G8. Scale bars: 2,000 μm. (**B**) Analysis of serum differential positive and negtive metabolites between 8-month WT and APP/PS1 mice. (**C**) KEGG enrichment analysis of different metabolites in serum between APP/PS1 and WT mice. (**D**–**M**) The differences in metabolites related to purine metabolism between the serum of APP/PS1 and WT mice (*n* = 8 per group). APP/PS1, APPswe/PSEN1dE9; KEGG, Kyoto Encyclopedia of Genes and Genomes. Data are presented as mean ± SD. Statistics were analyzed by two-tailed Student’s *t* test or rank sum tests based on the data’s normal distribution. *P < 0.05, **P < 0.01.

**Figure 2:**
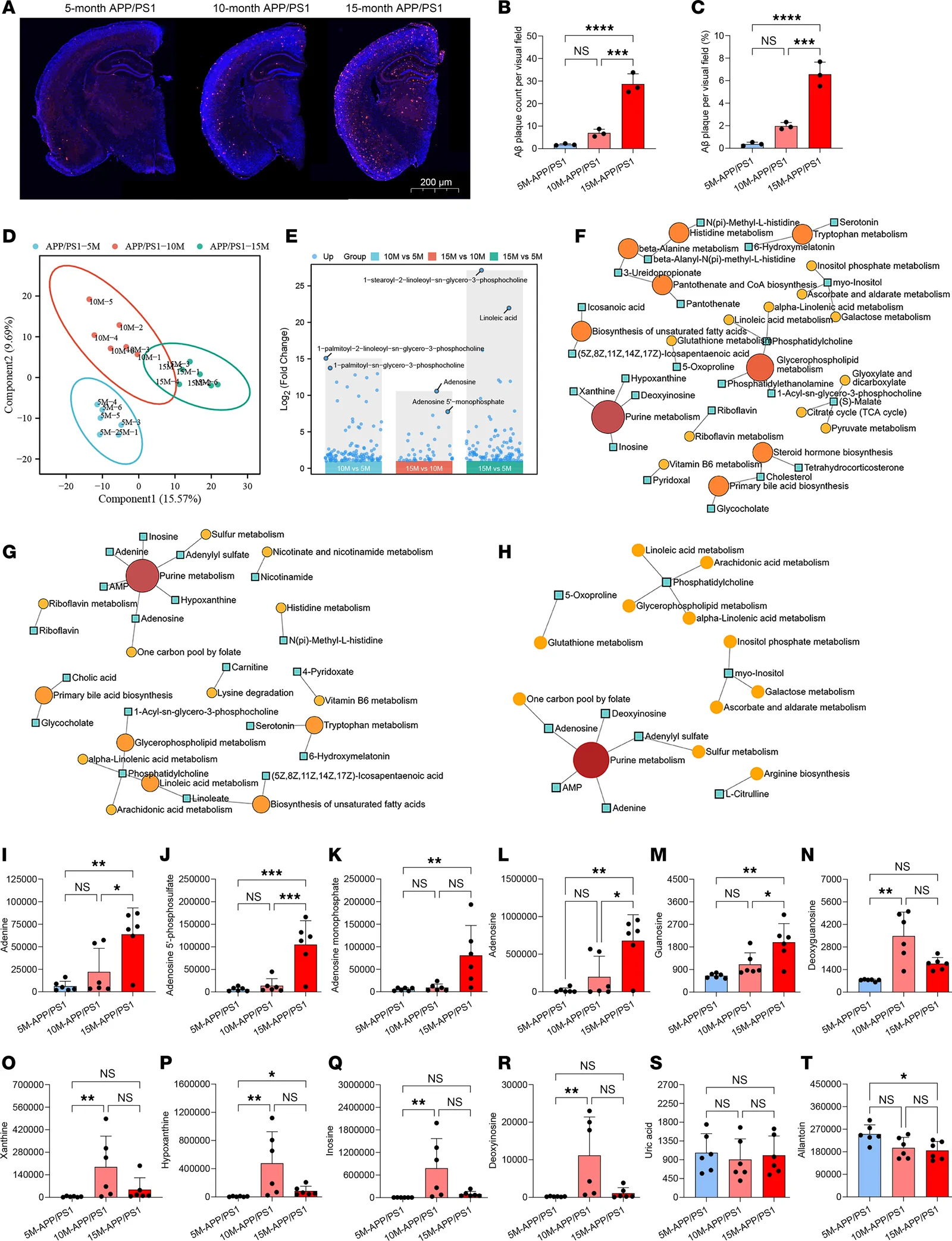
Metabolic profile differences in serum of APP/PS1 mice at different ages. (**A**–**C**) Immunofluorescence and quantification of 4G8. Scale bars: 2,000 μm (*n* = 3 per group). (**D**) PCA of metabolites in serum of 5-, 10-, and 15-month APP/PS1mice (*n* = 6 per group). (**E**) Analysis of serum differential metabolites between 5-, 10-, and 15-month APP/PS1mice. (**F**–**H**) KEGG enrichment analysis of different metabolites in serum between APP/PS1 and WT mice. (**I**–**T**) The differences in metabolites related to purine metabolism between the serum of APP/PS1 and WT mice. APP/PS1, APPswe/PSEN1dE9; PCA, Principal Component Analysis; KEGG, Kyoto Encyclopedia of Genes and Genomes. Data are presented as mean ± SD. Statistics were analyzed by Kruskal-Wallis test followed by Dunn’s multiple comparisons test or 1-way ANOVA followed by Tukey’s multiple comparisons test based on the normality of the data distribution. *P < 0. 05, **P < 0.01, ***P < 0.001, ****P < 0.0001.

**Figure 3:**
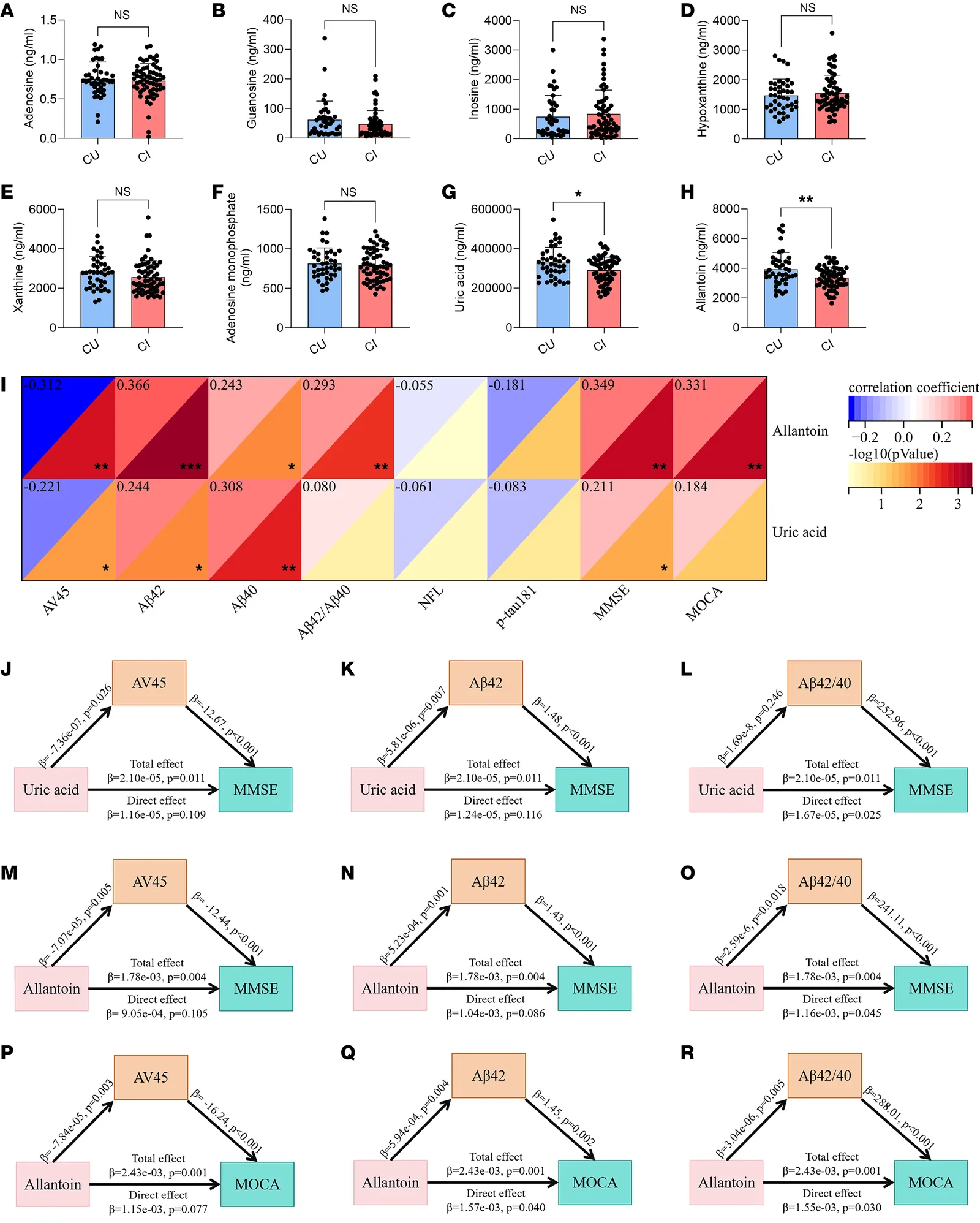
The difference in purine metabolism profile in the ADIMIC and their correlations with pathological markers. (**A**–**H**) The differences in metabolites related to purine metabolism between the serum of Aβ^+^ CI and Aβ^–^ CU group. Data are presented as mean ± SD. Statistics were analyzed using covariance adjusting for age, sex, education, and APOE4 carrier status. (**I**) Partial correlation analysis of the differences in purine metabolites with the AD biomarker standards and cognitive levels adjusting for age, sex, education, and APOE4 carrier status. (**J**–**L**) Mediating effect of AV45 global SUVR, Aβ42/40, and Aβ42 on association between uric acid and MMSE scores. (**M**–**O**) Mediating effect of AV45 global SUVR, Aβ42/40, and Aβ42 on association between allantoin and MMSE scores. (**P**–**R**) Mediating effect of AV45 global SUVR, Aβ42/40, and Aβ42 on association between allantoin and MOCA scores. All the mediation analysis were adjusted for age, sex, education, and APOE4 carrier status. Aβ, amyloid-β; ADIMIC, Alzheimer’s Disease Immunization and Microbiota Initiative study Cohort; CI, cognitively impaired; CU, cognitively unimpaired; MMSE, Mini-mental state examination, MOCA, Montreal cognitive assessment. *P < 0.05, **P < 0.01, ***P < 0.001.

**Figure 4:**
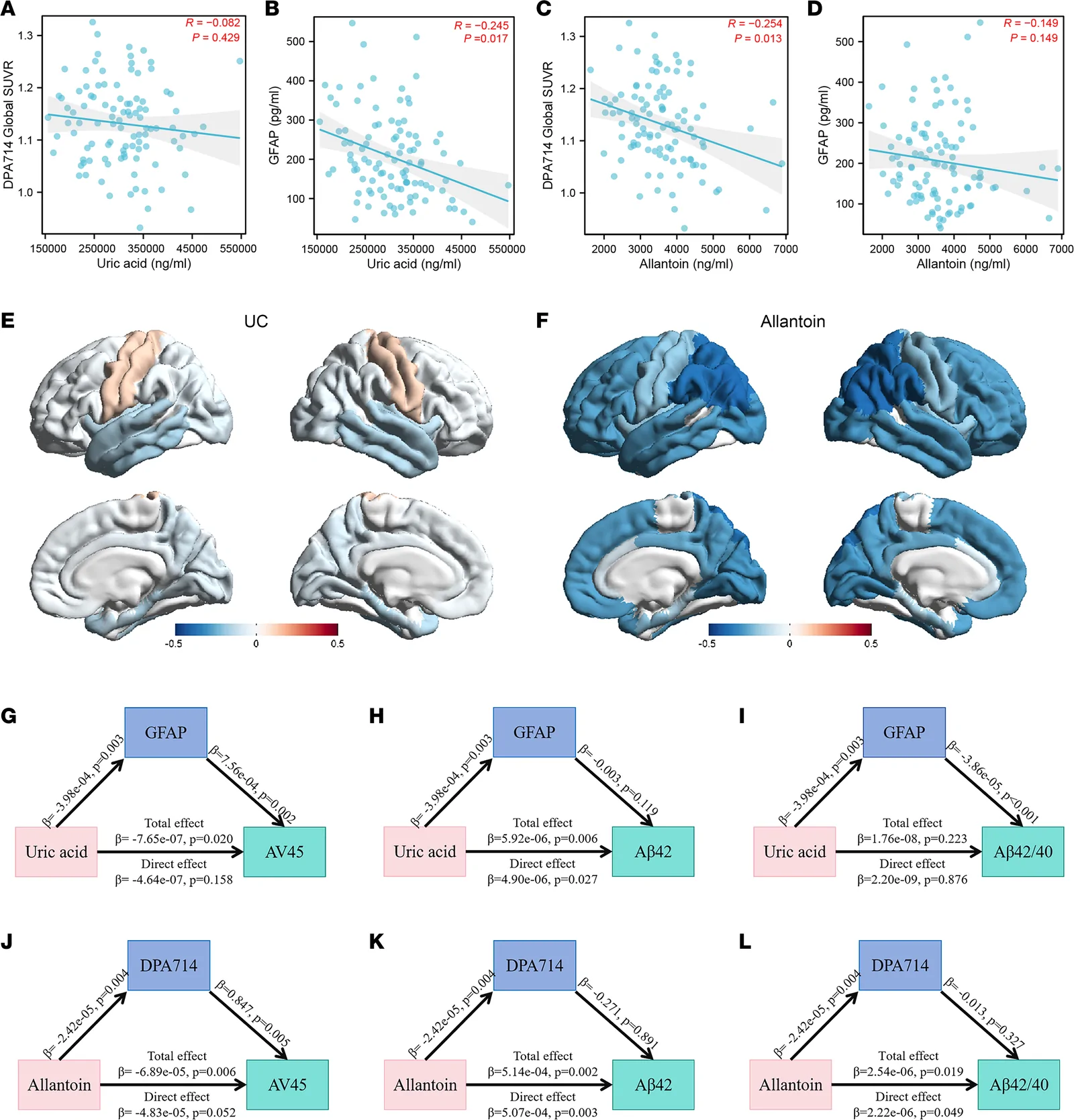
Uric acid and allantoin mediate Aβ pathology through neuroinflammation. (**A**) Partial correlation analysis between uric acid and DPA714 Global SUVR. (**B**) Partial correlation analysis between uric acid and plasma GFAP level. (**C**) Partial correlation analysis between allantoin and DPA714 Global SUVR. (**D**) Partial correlation analysis between allantoin and plasma GFAP level. (**E**) Brain region correlation between uric acid and DPA714 SUVR. (**F**) Brain region correlation between allantoin and DPA714 SUVR. (**G**–**I**) Mediating effect of GFAP on association between uric acid with AV45 global SUVR, Aβ40, and Aβ42. (**J**–**L**) Mediating effect of DPA714 global SUVR on association between allantoin with AV45 global SUVR, Aβ42, and Aβ42/40. All the analyses were adjusted for age, sex, education, and APOE4 carrier status. Aβ, amyloid-β; GFAP, glial fibrillary acidic protein; SUVR, Standard uptake value ratios.

**Figure 5:**
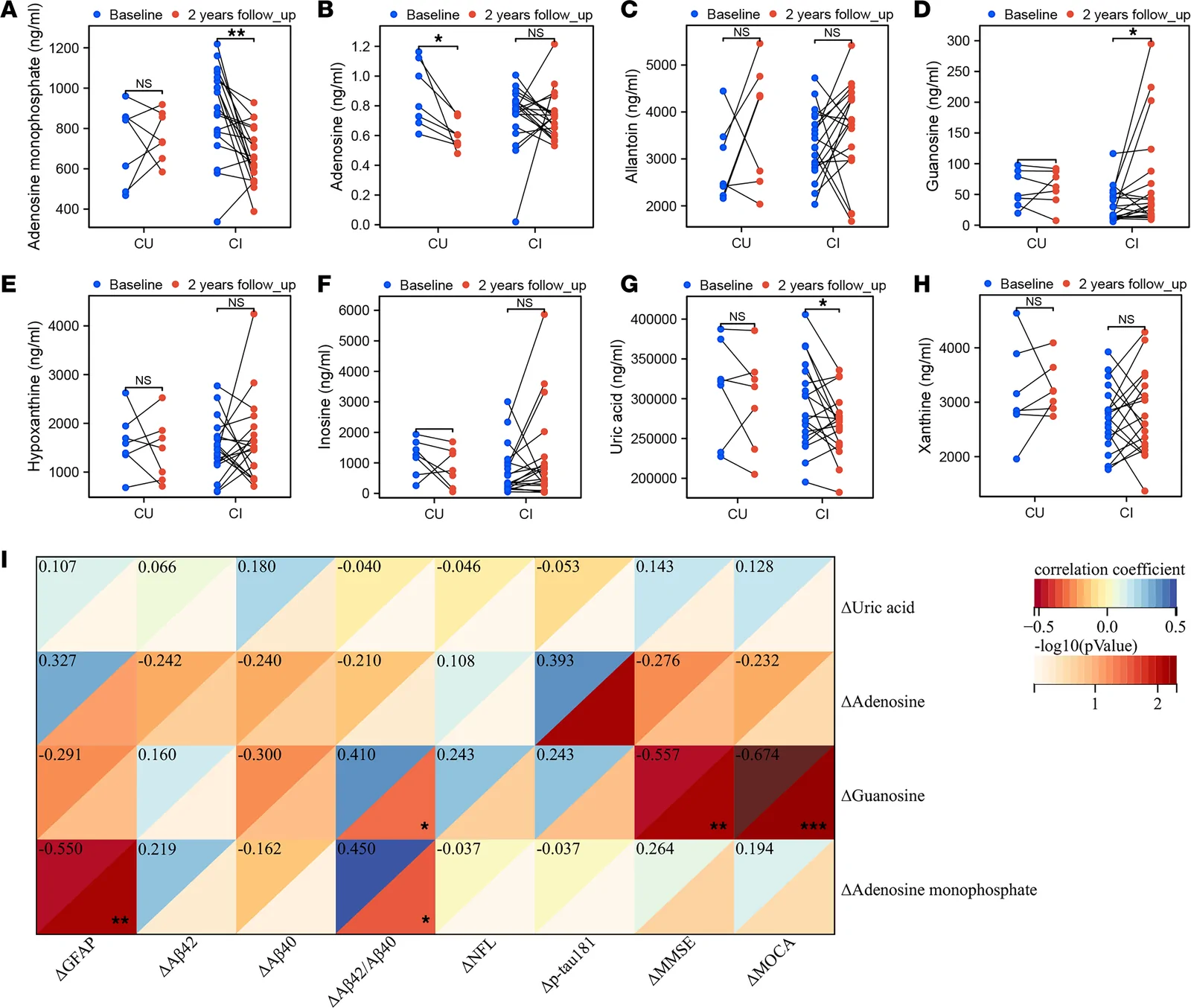
The longitudinal changes of purine metabolism and their correlation with the changes of pathological markers in ADIMIC. (**A**–**H**) Longitudinal data analysis of the longitudinal changes in purine metabolites. (**I**) Partial correlation analysis between longitudinal changes in purine metabolites and longitudinal changes in pathological markers adjusting for age, sex, education, and APOE4 carrier status. ADIMIC, Alzheimer’s Disease Immunization and Microbiota Initiative study Cohort. *P < 0.05, **P < 0.01, ***P < 0.001.

**Figure 6:**
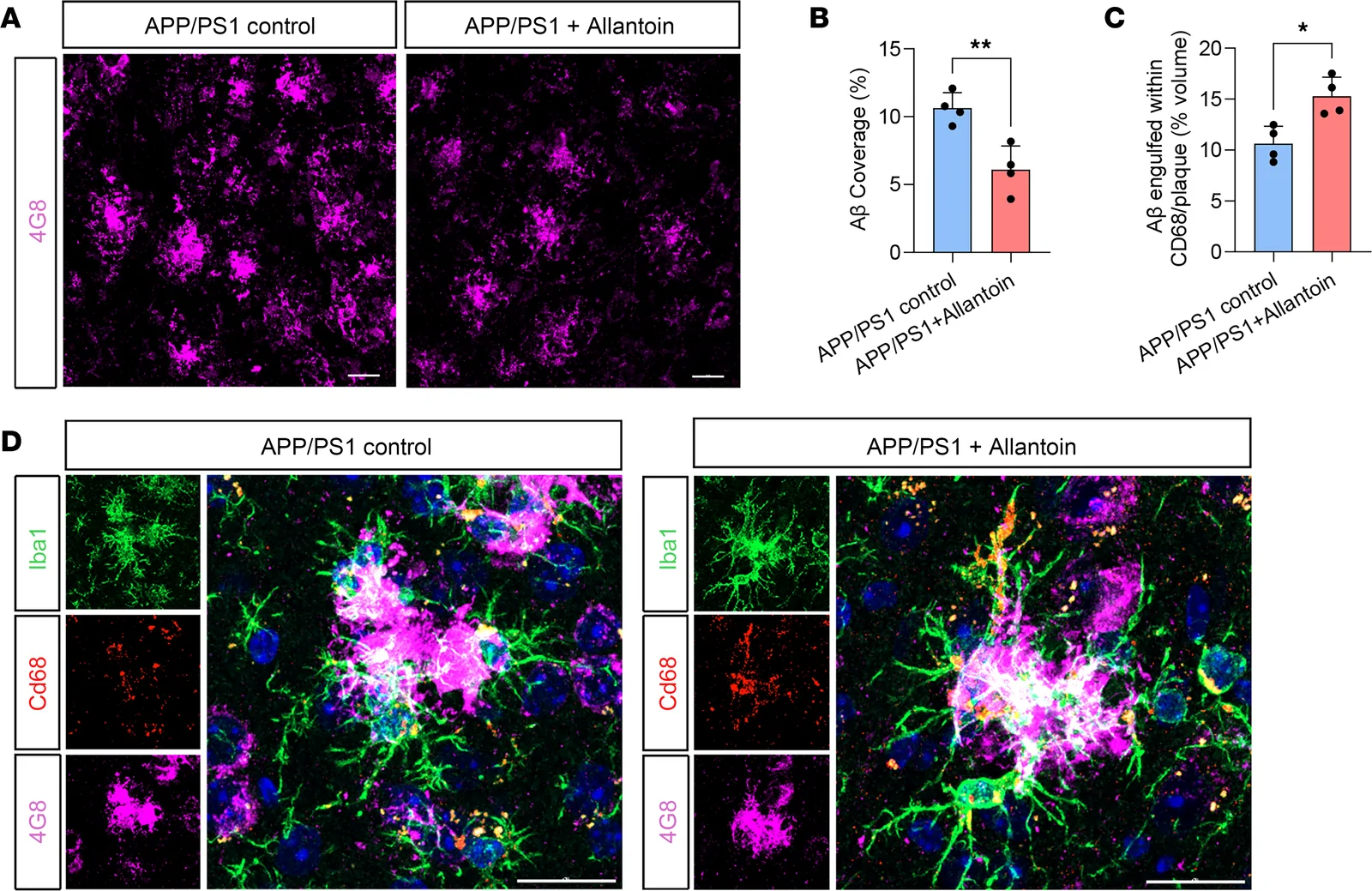
Allantoin supplementation reduced Aβ pathology through promoting microglial phagocytosis of Aβ in APP/PS1 mice. (**A** and **B**) Immunofluorescence and quantification of 4G8 in hippocampal subiculum. (**C** and **D**) Immunofluorescence and quantification of 4G8, Iba1, and Cd68 in hippocampal subiculum. *n* = 4 per group. Data are presented as mean ± SD. Statistics were analyzed using 2-tailed Student’s *t* test. *P < 0.05, **P < 0.01. Aβ, amyloid-β; APP/PS1, APPswe/PSEN1dE9. Scale bar: 20 μm.

**Table 1:**
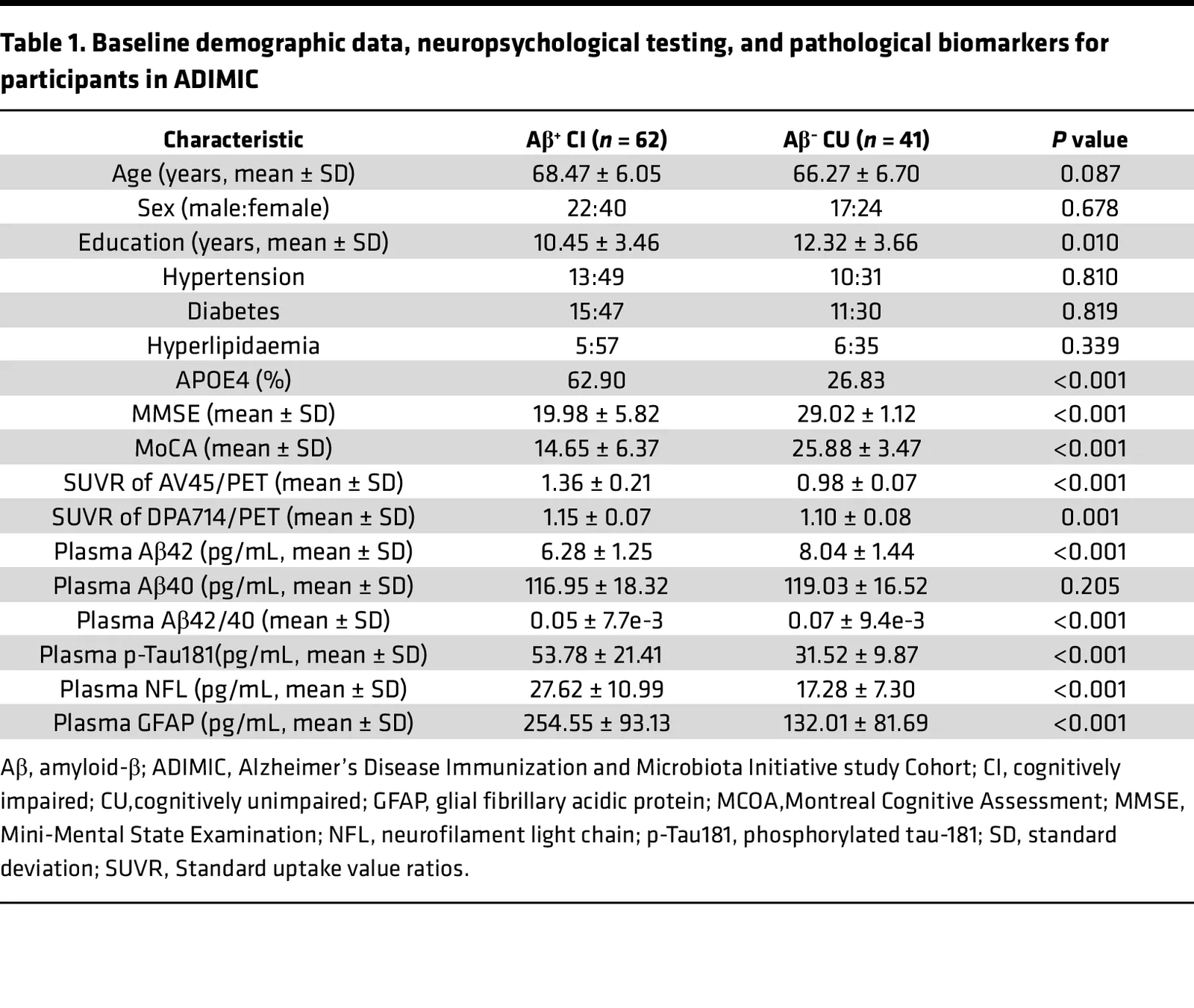
Baseline demographic data, neuropsychological testing, and pathological biomarkers for participants in ADIMIC

**Table 2:**
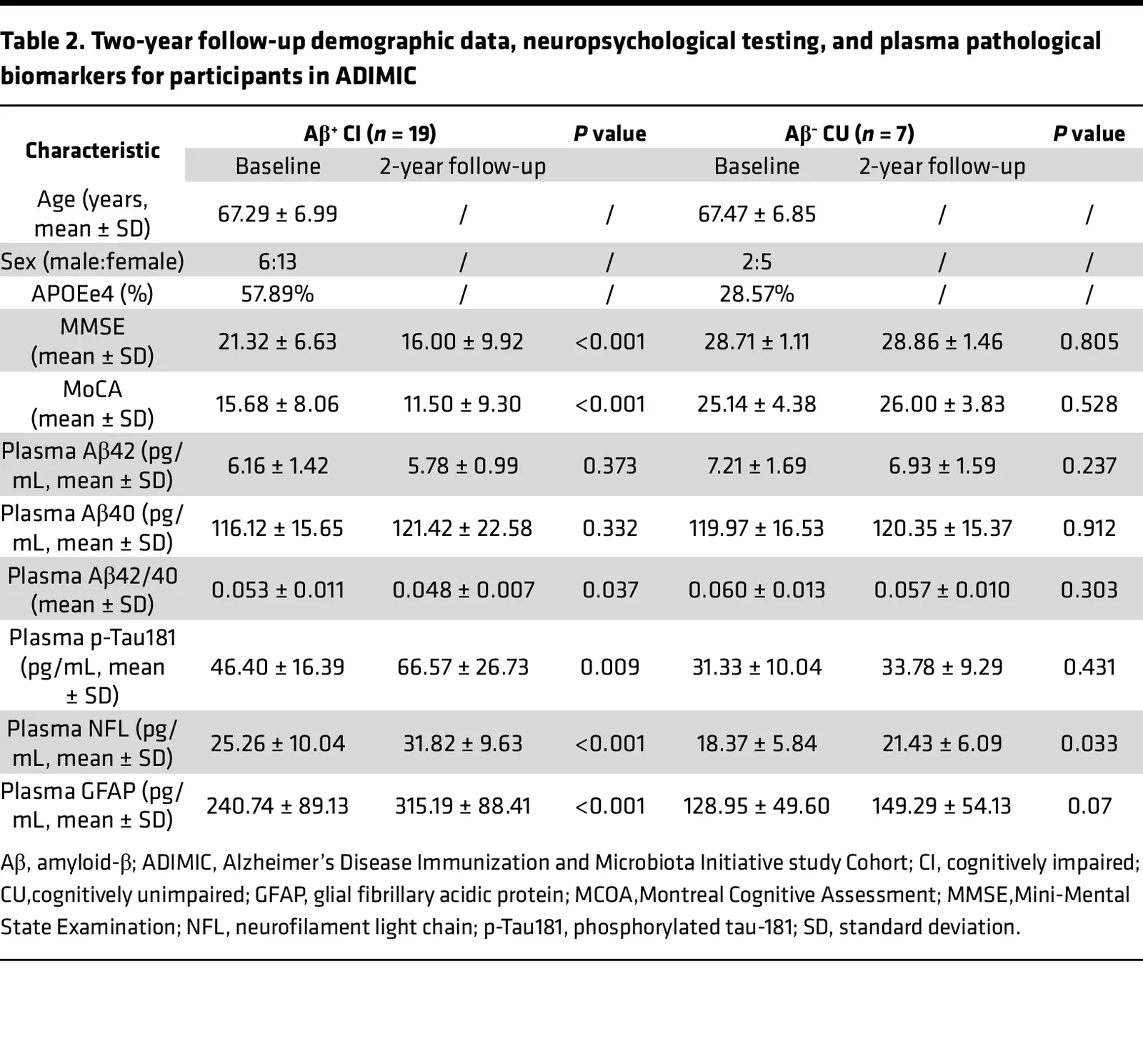
Two-year follow-up demographic data, neuropsychological testing, and plasma pathological biomarkers for participants in ADIMIC

## Version 1 10/22/2026

Electronic publication
